# A subunit vaccine based on *Brucella* rBP26 induces Th1 immune responses and M1 macrophage activation: Th1 and M1 activation by rBP26 *Brucella* vaccine

**DOI:** 10.3724/abbs.2024023

**Published:** 2024-02-29

**Authors:** Jia Wen, Zihua Li, Yongxue Lv, Shuqin Ding, Yazhou Zhu, Jihui Yang, Jing Tang, Mingxing Zhu, Yinqi Zhao, Wei Zhao

**Affiliations:** 1 School of Basic Medicine Ningxia Medical University Yinchuan 750004 China; 2 General Medicine Department General Hospital of Ningxia Medical University Yinchuan 750004 China; 3 Ningxia Key Laboratory of Prevention and Control of Common Infectious Diseases Ningxia Hui Autonomous Region Yinchuan 750004 China

**Keywords:** BP26, M1 macrophage, recombinant protein vaccine, Th1 immune responses, immunogenicity

## Abstract

Brucellosis is a global zoonotic infection caused by *Brucella* bacteria, which poses a significant burden on society. While transmission prevention is currently the most effective method, the absence of a licenced vaccine for humans necessitates the urgent development of a safe and effective vaccine. Recombinant protein-based subunit vaccines are considered promising options, and in this study, the *Brucella* BP26 protein is expressed using prokaryotic expression systems. The immune responses are evaluated using the well-established adjuvant CpG-ODN. The results demonstrate that rBP26 supplemented with a CpG adjuvant induces M1 macrophage polarization and stimulates cellular immune responses mediated by Th1 cells and CD8 ^+^ T cells. Additionally, it generates high levels of rBP26-specific antibodies in immunized mice. Furthermore, rBP26 immunization activates, proliferates, and produces cytokines in T lymphocytes while also maintaining immune memory for an extended period of time. These findings shed light on the potential biological function of rBP26, which is crucial for understanding brucellosis pathogenesis. Moreover, rBP26 holds promise as an effective subunit vaccine candidate for use in endemic areas.

## Introduction

Brucellosis, a zoonotic infection caused by *Brucella* bacteria, poses a significant public health concern, affecting an estimated 3.5 billion people worldwide [1]. *Brucella* are transmitted primarily through close contact with the blood, feces, urine, and placenta of infected host animals. Infections can result in miscarriages in female animals and orchitis in males, leading to a decrease in reproductive capacity and compromising the quality and safety of animal products [2]. In humans, brucellosis manifests as symptoms such as fever, arthritis, orchitis, hepatitis, encephalomyelitis, and endocarditis [3]. Presently, several live attenuated vaccines, including S19, Rev.1, S2, SR82, and RB51, are widely used [ 4, 5]; however, these vaccines have certain limitations. For instance, some infections and complications may occur in humans, including the risk of miscarriage in pregnant cows [6]. Given the absence of a licenced vaccine for human use, there is an urgent need to develop a safe and effective vaccine suitable for high-risk workers and individuals residing in endemic areas.

With advancements in molecular biology technology and a deeper understanding of the pathogenesis of brucellosis, there is a promising opportunity for the development of new transgenic vaccines to replace conventional vaccines for brucellosis control. Moreover, recombinant protein vaccines based on antigen recognition have gained significant attention and have been extensively studied [ 7, 8]. These vaccines, expressed in either eukaryotic or prokaryotic systems, offer the advantage of inducing high levels of antibodies while addressing safety concerns associated with live vaccines [6]. Researchers have cloned various *Brucella* outer membrane proteins and explored their immunogenicity in an effort to identify components that can effectively protect target animal species. Notable examples include p39 [9], OMP31 [10], Cu-Zn-SOD [11], L7/L12 [12], OMP2b [13], and TF [14]. These studies aimed to identify specific protein components that could be utilized in the development of protective vaccines. Among the many outer membrane proteins, BP26 is located in the periplasm of *Brucella* and has been identified as an important diagnostic antigen for brucellosis. It is highly conserved in the *Brucella* genus and widely used as a specific marker for diagnosing brucellosis [ 15‒ 17]. As a potential vaccine candidate, BP26 can stimulate both humoral and cellular immune responses in the host in combination with other agents, providing protection against *Brucella* infection [ 18, 19]. However, macrophages, which are the first line of defense against *Brucella*, play a crucial role in linking innate and adaptive immunity. When being infected with *Brucella*, macrophages recognize, bind, and internalize the pathogen while also secreting soluble antimicrobial agents and innate immune mediators to defend against the infection. Although several *Brucella* outer membrane proteins have been shown to activate macrophages to secrete pro-inflammatory cytokines, the function of rBP26 in innate and adaptive immunity, as well as its protective role in host defense against brucellosis, remains largely unknown.

To ensure the safety and effectiveness of the vaccine, we selected CpG as the adjuvant for our next study. CpG-ODN is a synthetic oligodeoxyribonucleotide that contains non-methylated cytosine guanine dinucleotide. It mimics the activation of cellular DNA and has been shown to have potent adjuvant activity when co-administered with antigens, stimulating cell-mediated immune responses [20]. Notably, CpG-ODNs have already been licenced as adjuvants for human hepatitis B virus vaccines, ensuring their safety.

In the present study, we successfully expressed rBP26 using a prokaryotic system. We then carried out further studies to investigate the effects of rBP26 on various aspects of the immune response, including macrophage polarisation, T lymphocyte activation and proliferation, and immune memory after immunisation. These findings provide a crucial theoretical foundation for better understanding the biological functions of BP26 and developing effective strategies for controlling brucellosis.

## Materials and Methods

### Animals

Female C57BL/6N mice aged 6‒8 weeks were obtained from Beijing Vital River Laboratory Animal Technology Co., Ltd (Beijing, China). The animal study protocol was approved by the Ethics Committee of the General Hospital of Ningxia Medical University (KYLL-2022-0685). All animals were administered isoflurane anaesthesia and subsequently euthanized through cervical dislocation.

### Chemicals and reagents

All the reagents used in the present study were of analytical and molecular biology grade. CpG-ODN 1826 (TCCATGACGTTCCTGACGTT) was synthesized by Sangon (Shanghai, China) and dissolved in sterile phosphate-buffered saline (PBS; 0.01 M, pH 7.2).

### PCR amplification and cloning

The *bp26* gene was amplified using *Brucella suis* S2 DNA as a template, and the resulting PCR product was inserted into the pET-30a vector after digestion using both the *EcoR*I and *Hind*III restriction enzymes, cloned and inserted into the pET-30a expression vector. The primers used in this study are shown as follows: forward, 5′-CGGAATTCATGAACACTCGTGCTAGCAATTTTCTC-3′ and reverse, 5′-CCCAAGCTTTTACTTGATTTCAAAAACGACATTGACCGA-3′. PCR was conducted for 35 cycles at 98°C for 2 min, 98°C for 20 s, 55°C for 30 s, 72°C for 30 s and finally 72°C for 5 min. The PCR amplification products were eluted from the agarose gel and ligated to the pET-30a vector using T4 DNA ligase. Finally, the newly constructed plasmid was transformed into *E*. *coli* BL21 (DE3) cells.

### Purification of rBP26 by Ni-NTA column chromatography

*E*. *coli* BL21 (DE3) carrying the recombinant plasmid pET30a- *bp26* was cultured in LB medium until the OD _600 nm_ reached 0.5. The cells were then induced with IPTG (0.5 mM) and incubated for an additional 12 h at 29°C. A His purification kit (Merck, Kenilworth, USA) was used to purify the rBP26 protein according to the manufacturer’s instructions. Purified rBP26 was identified by sodium dodecyl sulphate-polyacrylamide gel electrophoresis (SDS-PAGE), and the protein concentration was determined using a Bradford kit (Beyotime, Shanghai, China).

### Immunization

**Figure 1:**
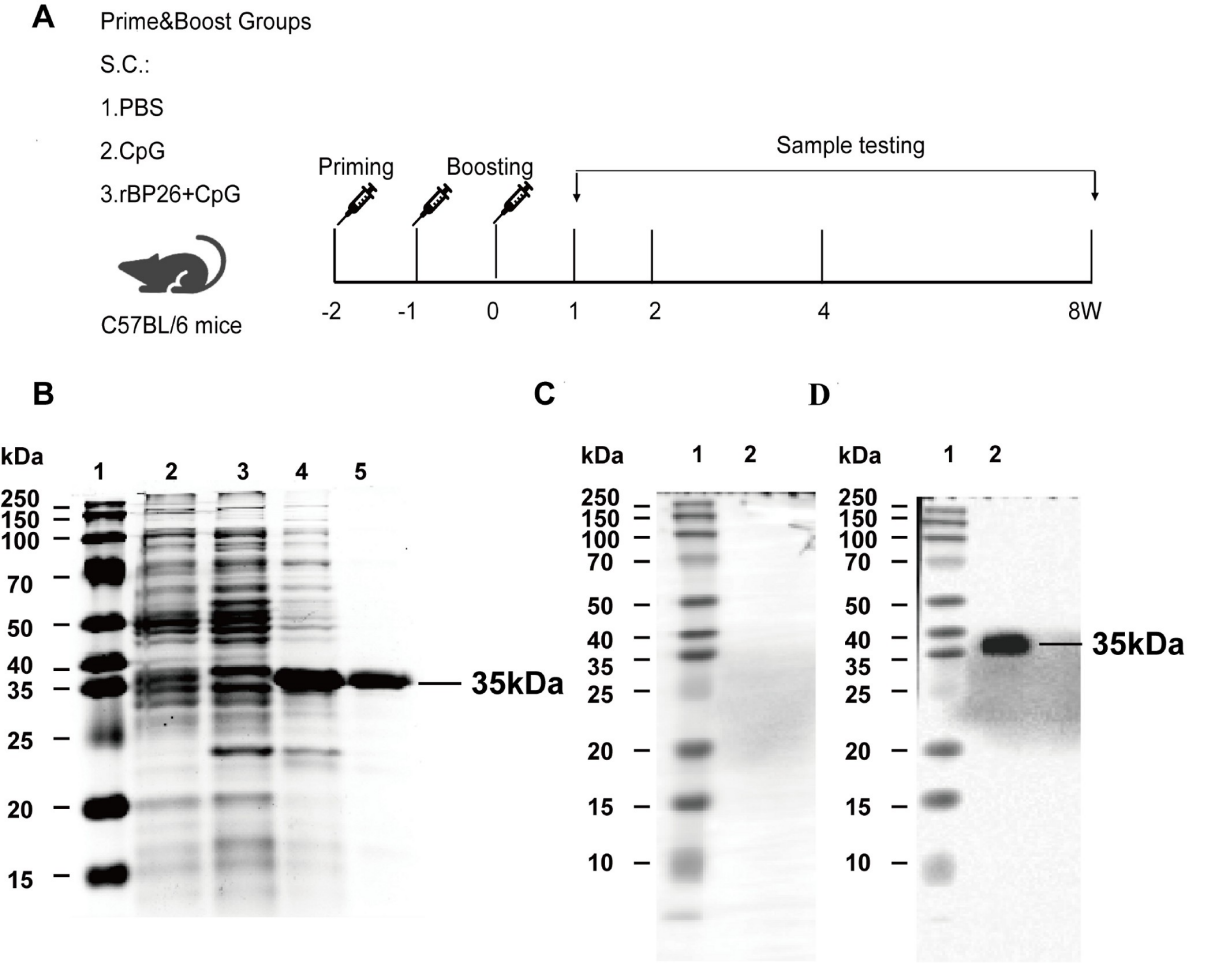
Figure 1 SDS-PAGE and western blot analysis of rBP26 and animal immunisation and sampling protocols (A) C57BL/6 mice (n=5) were subcutaneously immunized according to the prime-boost protocol. Two enhancements (‒1 and 0) were performed. Spleen and serum samples were collected and tested at 1, 2, 4 and 8 weeks after the last immunisation. (B) SDS-PAGE analysis of recombinant protein BP26. Lane 1, protein marker; Lane 2, empty vehicle induction product; Lane 3, uninduced negative control; Lane 4, whole bacteria after induction; and Lane 5, purfied rBP26. (C) Western blot analysis of rBP26 with serum from PBS immunised groups of mice. Lane 1, protein marker; and Lane 2, serum from mice in the PBS group. (D) Western blot of rBP26 with serum from rBP26 immunised groups of mice. Lane 1, protein marker; and Lane 2, serum from mice immunized with purified rBP26.

Here we established an immunological system using inbred female C57BL/6N mice. Fifteen mice were randomly divided into three groups: the control group (injected with PBS only), the CpG group (injected with 40 μg of CpG-ODN1826), and the rBP26+CpG group (injected with 30 μg of rBP26 and 40 μg of CpG-ODN1826). The mixture was then suspended in PBS and injected into the lower abdomen of the mice (100 μL/mouse). To boost immunity, purified rBP26 was mixed with CpG ODN 1826 as a protein vaccine. We performed two boosts, as shown in Figure 1A, one week apart.

### Sample collection and cell culture

At the designated time after immunization, the mice were anaesthetized with isoflurane, and blood and spleen samples were collected from each mouse. Each spleen was minced and filtered through a 70-μm mesh filter. Approximately 5 mL of erythrocyte lysis buffer was then added to the cell suspension, which was subsequently incubated on ice for 5 min. The reaction was stopped by adding an equal volume of PBS. The cells were subsequently centrifuged at 350 *g* for 5 min, washed once with PBS, and resuspended in complete RPMI-1640 medium (HyClone, Logan, USA) supplemented with 10% heat-inactivated fetal calf serum (FCS; GeminiBio, West Sacramento, USA), 100 μg/mL streptomycin, 100 U/mL penicillin, 2 mM L-glutamine, and 50 μM 2-mercaptoethanol (Gibco, Big Island, USA).

### Western blot analysis

The protein concentration was determined using a Bradford kit. The proteins were subsequently boiled at 100°C for 10 min and separated by using 12% SDS-PAGE. Polyvinylidene fluoride (PVDF) membranes (Solarbio Science & Technology Co., Ltd, Beijing, China) were used for protein transfer. The first antibody used for the rBP26 protein was obtained from the serum of immunized and control mice (1:500 dilution), and the second antibody used was goat anti-mouse IgG/HRP (1:20,000; Abcam, Shanghai, China). Finally, protein expression was analysed using an Enhanced chemiluminescence (ECL) detection kit (KeyGen Biotech Co., Ltd, Nanjing, China) on a ChemiDocTM touch imaging system (Bio-Rad, Shanghai, China).

### ELISA and ELISpot

To begin the experiment, 200 μL of splenocytes at a concentration of 2×10 ^6^ cells/mL were inoculated into 96-well plates. These plates were divided into two groups: one group was treated with recombinant BP26 at a concentration of 10 μg/mL, and the other group was not treated. Additionally, an anti-CD28 antibody was added to each well at a concentration of 1 μg/mL. The plates were then placed in an environment at 37°C with CO _2_ 5% concentration for 72 h. After the incubation period, the culture supernatant was collected, and the levels of cytokines (IFN-γ, TNF-α, IL-2, IL-4, and IL-6) were measured using a BD OptEIA ELISA kit (BD Biosciences, San Jose, USA). To stop the reaction, 1 M H _2_SO _4_ was added to each well. The OD value of each well was then determined at a wavelength of 450 nm using a microplate reader (Thermo Fisher, Waltham, USA).

A BD OptEIA Mouse ELISPOT kit (BD Biosciences) was used for the detection of IFN-γ and IL-4 puncta. First, the capture antibodies were diluted 1:250 in coating buffer, and the ELISPOT plates were diluted with 100 μL of capture antibody per well. The plate was then sealed and incubated at 4°C overnight. After the coated antibody was discarded, the wells were washed with 200 μL of Closure Solution (RPMI 1640 medium containing 10% fetal calf serum). Subsequently, 200 μL of Closure Solution was added to each well, and the plates were incubated at 37°C for 2 h. The cells were suspended in complete RPMI 1640 medium at a density of 1×10 ^6^ cells/mL and were incubated in ELISPOT plates at 200 μL per well. This mixture was incubated at 37°C and 5% CO _2_ in the presence of anti-CD28 (1 μg/mL) with or without rBP26 (10 μg/mL). The plates were subsequently washed twice with deionized water and three times with PBST. The detection antibody was diluted in dilution buffer (PBS containing 10% fetal calf serum) and incubated for 2 h at 37°C. This was achieved by adding 100 μL of diluted detection antibody to each well. The plate was then washed three times with PBST. Next, a predetermined dilution of HRP-conjugated antibodies in 100 μL of dilution buffer was added to each well, and the plate was incubated for 1 h at 37°C. The plates were washed four times with PBST and twice with PBS. Subsequently, 100 μL of 3-amino-9-ethylcarbazole (AEC) was added to each well. The substrate reaction was stopped using deionized water, and the spots were analyzed using an ELISPOT plate reader (AID Fluorospot; Autoimmun Diagnostika GmbH, Strassberg, Germany).

### Flow cytometric analysis

To assess intracellular cytokine levels, cells were cultured at a concentration of 1×10 ^6^ cells/mL in medium supplemented with an anti-CD28 antibody (1 μg/mL). The cells were then incubated with or without rBP26 at a concentration of 10 μg/mL for 24 h. During the last 6 h of incubation, brefeldin A (10 μg/mL; Sigma-Aldrich, St Louis, USA) was added. The cells were subsequently washed twice with PBS containing 0.1% BSA and 0.05% sodium (buffer 1) and stained with fluorescent dye-labelled monoclonal antibodies for 30 min at 4°C in the dark. This staining procedure was subsequently conducted for phenotypic analysis. After staining, the cells were washed with buffer 1 and fixed with 4% paraformaldehyde. Subsequently, the cells were permeabilized overnight at 4°C using buffer 2 (buffer 1 supplemented with 0.1% diosgenin). The cells were then stained with fluorescence-labelled monoclonal antibodies in the dark at 4°C for 30 min. Following another wash with buffer 1, the levels of intracellular cytokines were determined using the BD Biosciences FACSCelesta method. The data were analyzed using FlowJo 10 software (TreeStar, San Carlos, USA). Prior to analysing the macrophages, they were treated with the Fcg blocking antibody against mouse CD16/32 for 10 min, followed by the same steps as those used for the determination of intracellular factors.

The following fluorescein antibodies are purchased from BD Bioscience, including phycoerythrin-Texas red (PE-CF594)-coupled anti-CD3 antibody, allophycocyanin-7 (APC-Cy7)-coupled anti-CD4 antibody, Pacific blue (Pacific Blue)-coupled anti-CD8 antibody, fluorescein isothiocyanate (FITC)-coupled anti-interferon-γ antibody, brilliant Violet 605 (BV605)-coupled anti-IL-4 antibody, PE-coupled anti-CD25 antibody, Alexa Fluor-coupled anti-CD69 antibody, immobilized active antibody BV510, Block mouse anti-CD16/32, APC-coupled anti-CD44 antibody, PE-coupled anti-CD25 antibody, Alexa Fluor-coupled anti-CD69 antibody, immobilized active antibody BV510, FCG anti-CD16/32, APC-coupled anti-CD44 antibody, PE-coupled anti-CD25 antibody, Alexa Fluor-coupled anti-CD69 antibody, fixable active antibody BV510, Blocking anti-CD16/32Peridinin chlorophyll protein-Cyanine5.5 (PerCP-CY5.5) conjugated-anti-CD62L antibody, BV421 Rat anti-mouse F4/80, FITC conjugated-anti-CD11b antibody, PerCP-CY5.5 conjugated-anti-CD11c antibody, and Alexa Fluor647-conjugated-anti-CD206 antibody.

### Cell proliferation assay

Briefly, splenocytes were washed twice with prewarmed PBS, incubated with 2.5 μM carboxyfluorescein diacetate succinimide (CFSE; Invitrogen, Carlsbad, USA) for 15 min in the dark at 37°C, and then washed with precooled PBS (containing 10% FBS). The cells were washed twice with precooled phosphate buffer and cultured with or without rBP26 (10 μg/mL) for a period of time at 37°C and 5% carbon dioxide. The cells were harvested, and phenotypic analysis was performed using fluorescence-labelled monoclonal antibodies in the dark at 4°C. The samples were scored using FACSCelesta, and the data were analyzed using FlowJo.

### Statistical analysis

All the statistical tests were performed using GraphPad Prism 10.1 (GraphPad, San Diego, USA) and SPSS 22.0. A nonpaired *t* test was used for comparisons between groups, and one-way or two-way analysis was used for comparisons among more than two groups. Normally distributed or homogeneously distributed data were tested by the LSD test or SNK test. Dunnett’s *t* test or an independent sample *t* test was used to test normally distributed data with unequal variance. The data are expressed as the mean±standard deviation. *P*<0.05 was considered to indicate statistical significance.

## Results

### SDS-PAGE and immunoblot analysis of recombinant BP26

To obtain the purified recombinant protein, the pET30a expression vector system was used to facilitate the expression of the protein encoded by the *bp26* gene in *E*. *coli*. Following induction of the recombinant expression vector pET30a- *bp26*/BL21 (DE3) with IPTG, the BP26 protein was purified using affinity chromatography on a Ni-NTA column. The purity and integrity of the proteins were confirmed through analysis via SDS-PAGE. Remarkably, distinct and well-defined protein bands were observed at approximately 35 kDa ( Figure 1B).

To assess the antigenicity and specificity of the purified rBP26 protein, western blot analysis was conducted using antisera obtained from immunized mice. The rBP26 protein exhibited no reactivity with antisera derived from PBS-treated mice ( Figure 1C). Conversely, the protein was able to recognize antisera obtained from rBP26-immunized mice ( Figure 1D). This observation indicated that the sera of immunized mice contained specific antibodies capable of binding to the rBP26 protein, thereby demonstrating its immunogenicity.

### rBP26 induces stronger and longer lasting antibody responses when combined with CpG adjuvant

**Figure 2:**
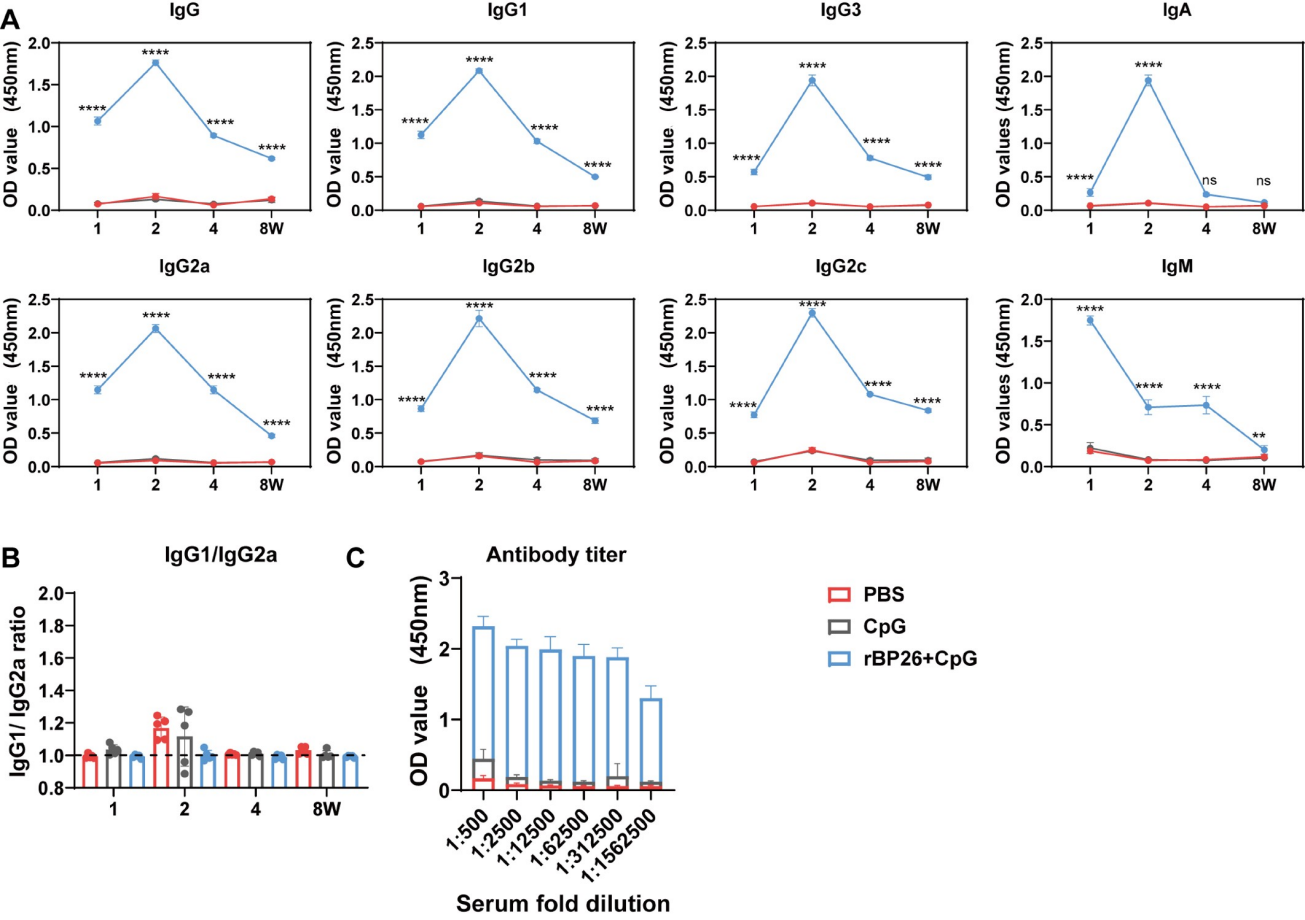
Figure 2 Antigen-specific humoral immunity induced after recombinant protein vaccination Serum samples were collected at 1, 2, 4 and 8 weeks after last immunisation and antibody levels were determined. (A) rBP26-specific antibodies. (B) rBP26-specific IgG1/IgG2a ratio. (C) Titers of rBP26-specific IgG antibodies in serum from C57BL/6 mice were detected. Data were collected from a sample size of 5 mice, and the error bars indicate the standard deviation. ****P<0.0001. ns, P>0.05.

Serum samples were collected at specific time points (1, 2, 4, and 8 weeks) after the final immunization to assess the presence of specific antibodies against rBP26 using an indirect ELISA. The antibodies tested included IgM, IgG, IgG1, IgG3, IgG2a, IgG2b, IgG2c, and IgA. As anticipated, immunization with rBP26 resulted in significantly greater levels of rBP26-specific IgG antibodies and their subtypes than did immunization with the control agents (PBS and CpG). The peak antibody response for most subtypes, except for IgM, was observed two weeks after the final immunization and remained detectable for at least 8 weeks. Notably, the expression level of IgA in the rBP26-immunized group reached its highest point after two weeks of booster immunization, but the duration was relatively short ( Figure 2A).

Furthermore, the analysis of Th1 (IgG2a) and Th2 (IgG1) antibody levels revealed a significant increase in the immunized group compared to the PBS and CpG control groups. The IgG1/IgG2a ratio, measured at detection, was consistently less than 1, indicating that rBP26 immunization primarily induced a Th1 immune response ( Figure 2B). Additionally, the antibody titres produced by rBP26 immunization were greater than those observed in the PBS and CpG groups ( Figure 2C). These findings strongly suggest that mice vaccinated with CpG-adjuvanted rBP26 exhibit robust and sustained antibody responses.

### Antigen-specific cellular immune responses to the rBP26 vaccine

**Figure 3:**
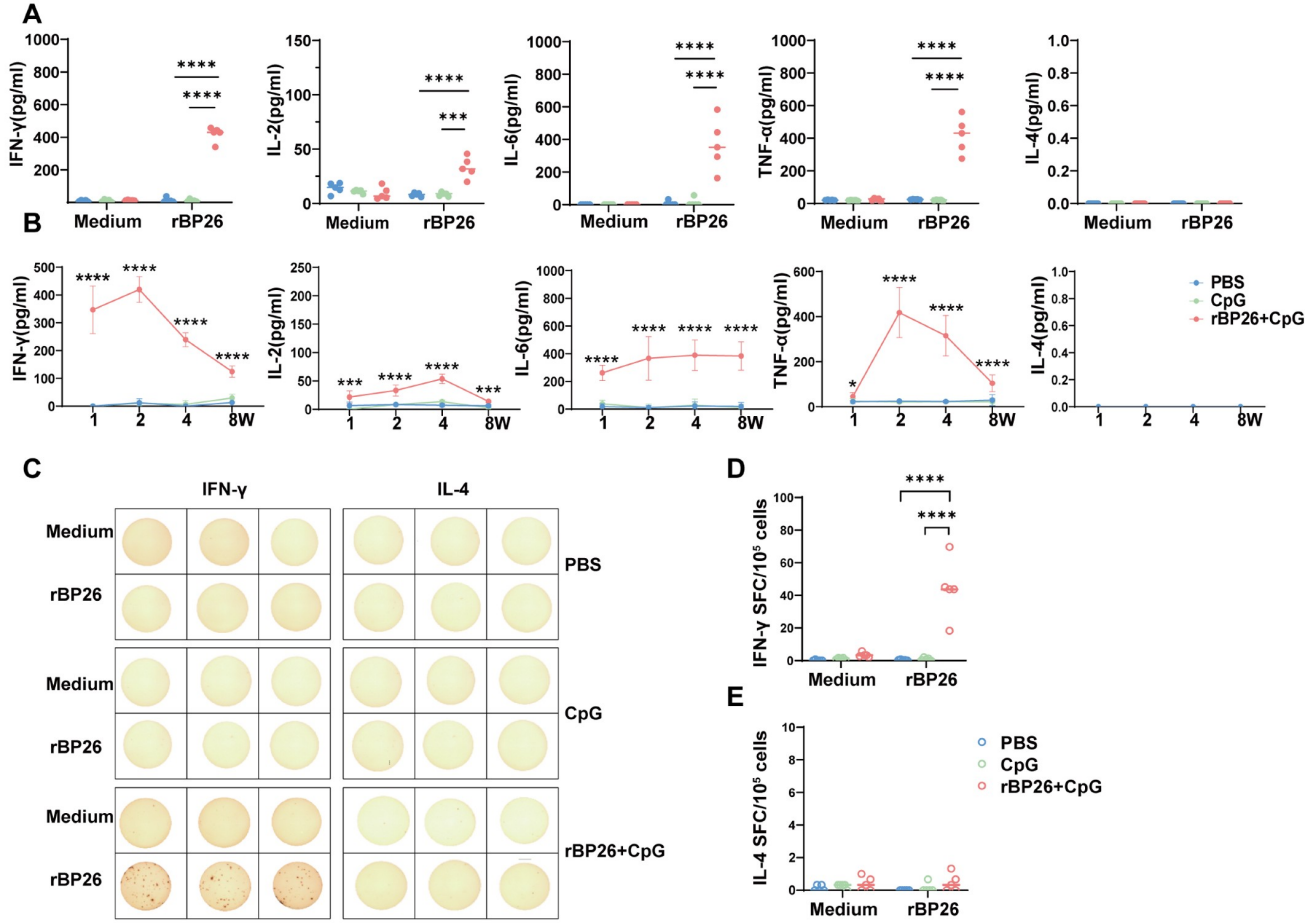
Figure 3 Ability of rBP26 to stimulate cytokine production Mice from different group were primed and boosted with PBS, CpG, or rBP26+CpG, respectively, and sacrificed at week 2 after boosting. Splenocytes were isolated from the spleen tissues. (A) At 2 weeks after booster immunization, splenocytes were isolated and stimulated with rBP26, and cytokine production of the supernatant was tested by ELISA. (B) At 1, 2, 4 and 8 weeks post-immunization, splenocytes were collected, and cytokine levels were determined by ELISA. (C) Splenocytes after 1 week post-immunization were collected, stimulated with rBP26 in vitro, and antigen-specific IFN-γ and IL-4 secretion were measured using ELISPOT. (D) Antigen-specific IFN-γ spot-forming cells. (E) Antigen-specific IL-4 spot-forming cells. Data were collected from a sample size of 5 mice, and the error bars indicate the standard deviation. ***P<0.001, ****P<0.0001. ns, P>0.05.

At 2 weeks after the final immunization, the levels of IFN-γ, IL-2, TNF-α, and IL-6 secreted by splenocytes in the rBP26-immunized group were significantly greater than those in the PBS group and the CpG adjuvant group following *in vitro* rBP26 stimulation. Conversely, the secretion of IL-4 did not significantly differ among the three groups ( Figure 3A). These findings strongly suggest that rBP26 immunization primarily elicits Th1-type cellular immune responses rather than Th2-type cellular immune responses. Furthermore, the secretion levels of IFN-γ and TNF-α in the rBP26-immunized group peaked approximately two weeks after the final immunization and gradually declined thereafter until the eighth week. Notably, no IL-4 secretion was detected during this period ( Figure 3B).

To further confirm the effectiveness of the recombinant protein vaccine in stimulating cellular immune responses in mice, splenocytes were collected for IFN-γ/IL-4 ELISPOT analysis after 1 week of rBP26 immunization. The results demonstrated that the rBP26-immunized group had a greater number of rBP26-specific IFN-γ-expressing splenocytes than the PBS and CpG groups did ( Figure 3C,D). However, similar numbers of splenocytes specifically expressing IL-4 were observed in the rBP26, PBS, and CpG groups ( Figure 3C,E).

### rBP26 activates M1 macrophages as well as antigen-specific CD4 ^+^ and CD8 ^+^ T cells

**Figure 4:**
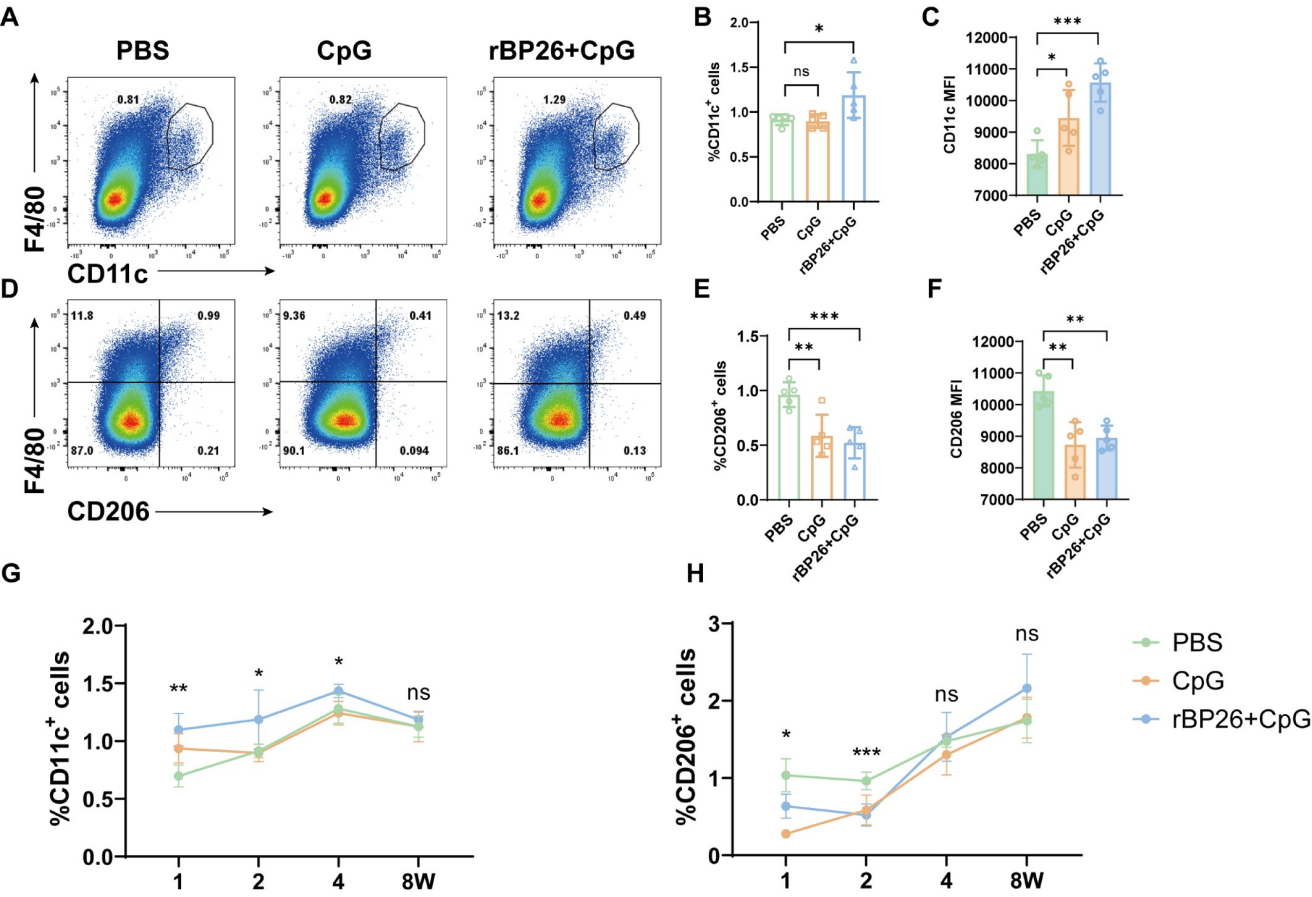
Figure 4 Detection of macrophage polarization through flow cytometry Splenocytes were isolated from mice injected with PBS, CpG, and rBP26+CpG at 1, 2, 4, and 8 weeks following the final immunization. The macrophage phenotype was analyzed using flow cytometry after surface labeling and intracellular staining. (A) Representative dot plot showed the identification of M1 macrophages (F4/80+ CD11c+ cells) at 2 week after the last immunisation. (B) Frequency of M1 macrophages. (C) MFI of CD11c. (D) Representative dot plot showed the identification of M2 macrophages (F4/80+ CD206+ cells) at 2 week after the last immunisation. (E) Frequency of M2 macrophages. (F) MFI of CD206. (G) Comparison of frequencies of M1 macrophages at different time interval. (H) Comparison of frequencies of M2 macrophages at different time interval. Data were collected from a sample size of 5 mice, and the error bars indicate the standard deviation. *P<0.05, **P<0.01, ***P<0.001. ns, P>0.05.

The immune cell profiles of mouse spleen tissues were assessed using flow cytometry analysis. Briefly, splenocytes were extracted from the spleens of mice in the PBS, CpG, and rBP26+CpG groups at 1, 2, 4, and 8 weeks after booster immunization. Flow cytometry was used to analyse the immune cell profiles of both M1 and M2 macrophages in detail ( Figure 4A,D). During the second week following the final immunization, compared to the control groups, the number of M1 macrophages (F4/80 ^+^CD11c ^+^ cells) and the mean fluorescence intensity (MFI) of CD11c in the rBP26-immunized group were significantly greater ( Figure 4B,C). Conversely, the number of M2 macrophages (F4/80 ^+^CD206 ^+^ cells) and the MFI of CD206 were significantly lower in the rBP26-immunized group ( Figure 4E,F). Furthermore, phenotypic changes in macrophages were observed at different time points after immunization. Specifically, compared with those in the PBS and CpG groups, the number of M1 macrophages in the rBP26-immunized group was significantly greater in the first and second weeks following immunization ( Figure 4G), while the number of M2 macrophages was significantly lower ( Figure 4H). However, there was minimal disparity in the number of M1-type and M2-type macrophages among the three groups at 4 and 8 weeks after immunization ( Figure 4G,H).

**Figure 5:**
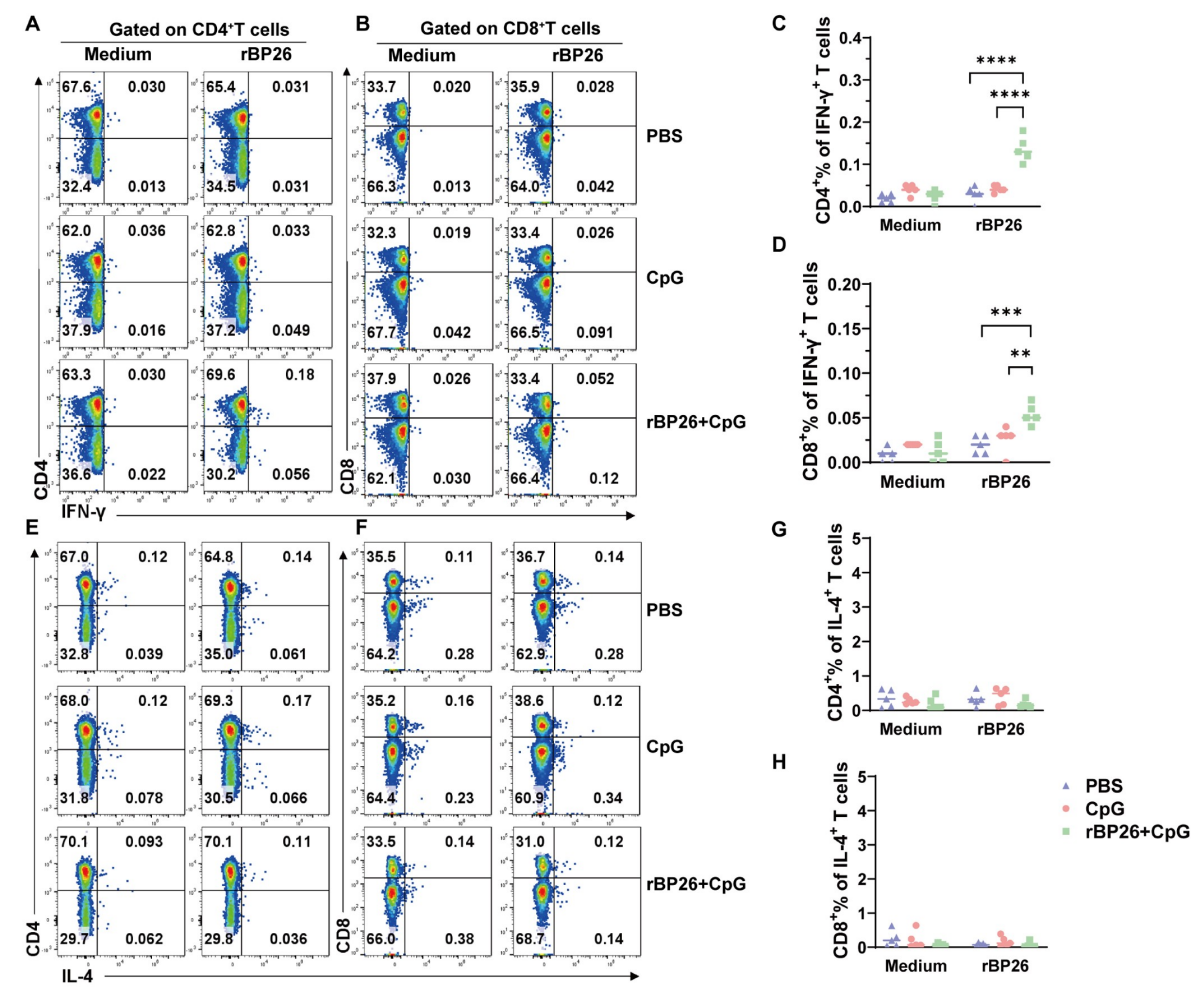
Figure 5 Flow cytometry analysis of antigen-specific CD4 ^+^ and CD8 ^+^ T-cell secretion of IFN-γ and IL-4 in splenocytes after immunization One week after the last immunisation, splenocytes were isolated, and stimulated with rBP26 or culture medium. Antigen-specific CD4+/CD8+ T-cell secretion of IFN-γ/IL-4 was determined using flow cytometry assay. Representative flow cytometry plots for measuring IFN-γ-secreting (A) CD4+ and (B) CD8+ T cells. Antigen-specific (C) CD4+ and (D) CD8+ T cells were analyzed for secretion of IFN-γ from the spleens. Representative flow cytometry plots for measuring IL-4-secreting (E) CD4+ and (F) CD8+ T cells. Antigen-specific (G) CD4+ and (H) CD8+ T cells were analyzed for secretion of IL-4 from the spleens. Data were collected from a sample size of 5 mice, and the error bars indicate the standard deviation. **P<0.01, ***P<0.001, ****P<0.0001.

To evaluate the potential disparities in the quality of T-cell responses in mice immunized with rBP26, a thorough analysis of antigen-specific T-cell responses was conducted. This analysis aimed to assess the presence of IFN-γ and IL-4-secreting T cells in an antigen-dependent manner. For this purpose, splenocytes collected from immunized mice were stimulated with rBP26 *in vitro*, and the resulting cytokine-positive T-cell subsets were subsequently analyzed using flow cytometry. Compared to those in the PBS and CpG groups, the frequency of CD4 ^+^ IFN-γ ^+^ T cells in the rBP26-immunized group was significantly greater ( Figure 5A,C), indicating a robust cellular immune response. However, there was no significant difference in the number of IL-4-secreting CD4 ^+^ T cells among the three groups ( Figure 5E,G). Furthermore, the frequency of IFN-γ-secreting CD8 ^+^ T cells also increased following stimulation with rBP26 compared to that in unstimulated splenocytes ( Figure 5B,D). Like in the CD4 ^+^ T-cell response, there was no notable difference in the frequency of CD8 ^+^ IL-4 ^+^ T cells among the different groups ( Figure 5F,H).

### rBP26 promotes the activation and proliferation of CD4 ^+^ and CD8 ^+^ T cells

**Figure 6:**
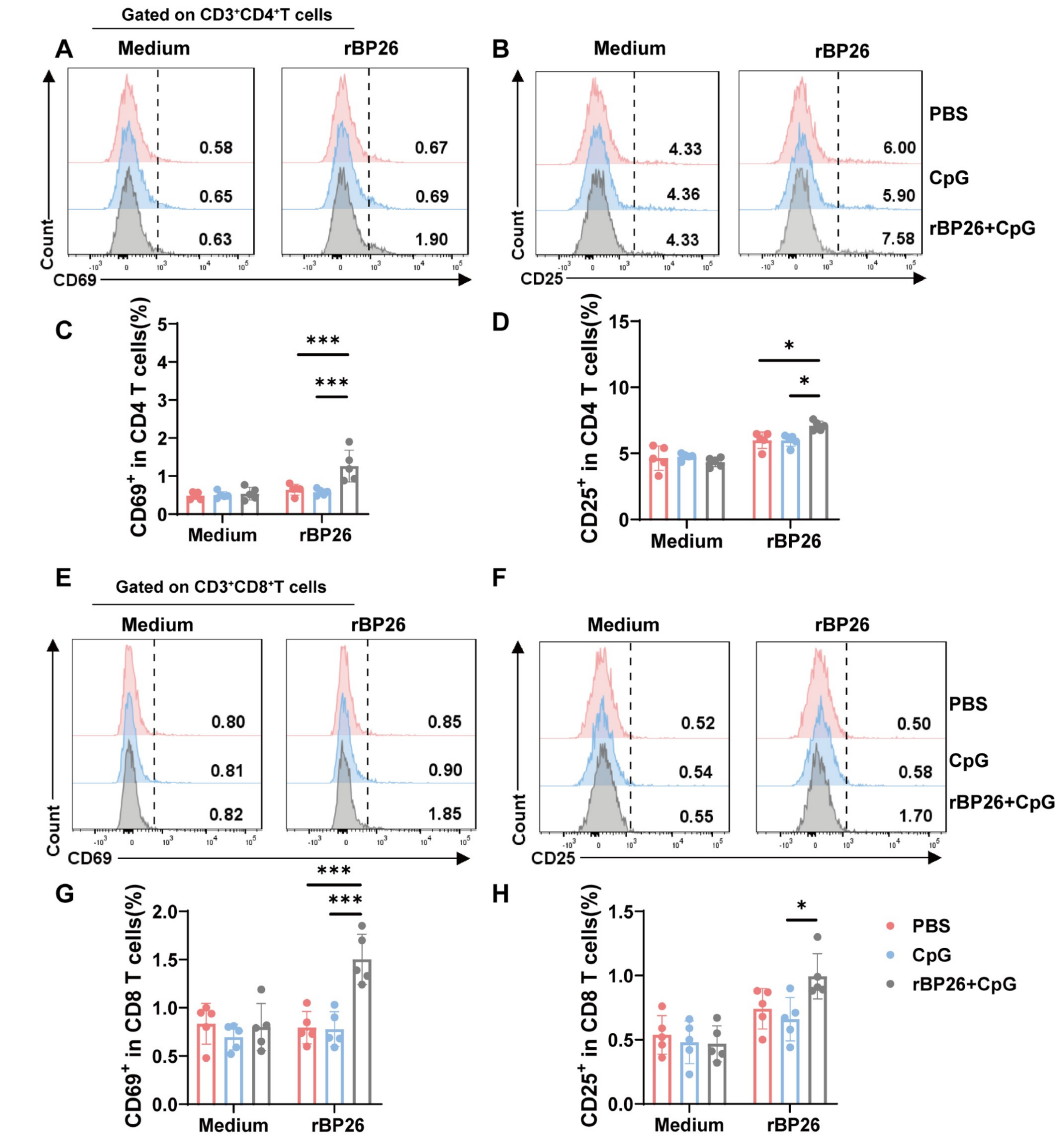
Figure 6 rBP26 activated specific CD4 ^+^ and CD8 ^+^ T cells Mice were induced and enhanced with PBS, CpG, or rBP26+CpG, and were executed at the second week after enhancement. Splenocytes were isolated and cultured with rBP26 or culture medium for 1 day. After stained with fluorochrome-conjugated monoclonal antibodies for CD69 and CD25, cells were analyzed by flow cytometry and data were analyzed using FlowJo. Representative peak plots showing (A) CD69 and (B) CD25 expressions in CD4+ T cells. Frequencies of (C) CD69 and (D) CD25 expression in CD4+ T cells from PBS, CpG, and rBP26+CpG group. Representative peak plots showing (E) CD69 and (F) CD25 expressions in CD8+ T cells. Frequencies of (G) CD69 and (H) CD25 expression in CD8+ T cells from PBS, CpG, and rBP26+CpG group. Data were collected from a sample size of 5 mice, and the error bars indicate the standard deviation. *P<0.05, ***P<0.001.

To gain more insight into the kinetics of T-cell activation, we examined the peak expression of CD69 and CD25 on CD4 ^+^ T cells ( Figure 6A,B) and CD8 ^+^ T cells by flow cytometry analysis ( Figure 6E,F). Following incubation with rBP26 (10 μg/mL) and an anti-CD28 antibody (1 μg/mL), we observed significantly greater CD69 and CD25 expression on CD4 ^+^ T cells in the rBP26-immunized group than in the PBS and CpG groups ( Figure 6C,D). Notably, CD8 ^+^ T cells also exhibited activation in the rBP26 groups ( Figure 6G,H).

**Figure 7:**
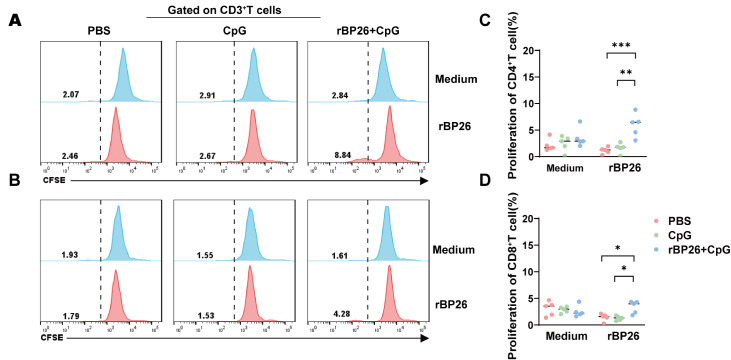
Figure 7 rBP26 promoted the proliferation of CD4 ^+^ and CD8 ^+^ T cells Mice were primed and boosted with PBS, CpG, or rBP26+CpG and sacrificed at week 2 after boosting. Splenocytes were treated with CFSE labelling and stimulated with rBP26 or culture medium for 5 days. Then cells were stained with fluorochrome-conjugated monoclonal antibodies for CD4 and CD8. Flow cytometry and FlowJo were performed to collect and analyze the data. Representative peak plots showing the identification of proliferative (A) CD4+ and (B) CD8+ T cells. Frequencies of proliferative (C) CD4+ and (D) CD8+ T cells. Data were collected from a sample size of 5 mice, and the error bars indicate the standard deviation. *P<0.05, **P<0.01, ***P<0.001.

To investigate cell proliferation, we employed CFSE labelling. As expected, the rBP26-immunized group displayed enhanced proliferation of CD4 ^+^ T cells on day 5 poststimulation with rBP26 in vitro ( Figure 7A,C). Similarly, rBP26 promoted the proliferation of CD8 ^+^ T cells ( Figure 7B,D). Importantly, this proliferation phenomenon was not observed in the PBS or CpG adjuvant groups.

### rBP26 vaccine induces splenic memory cells

**Figure 8:**
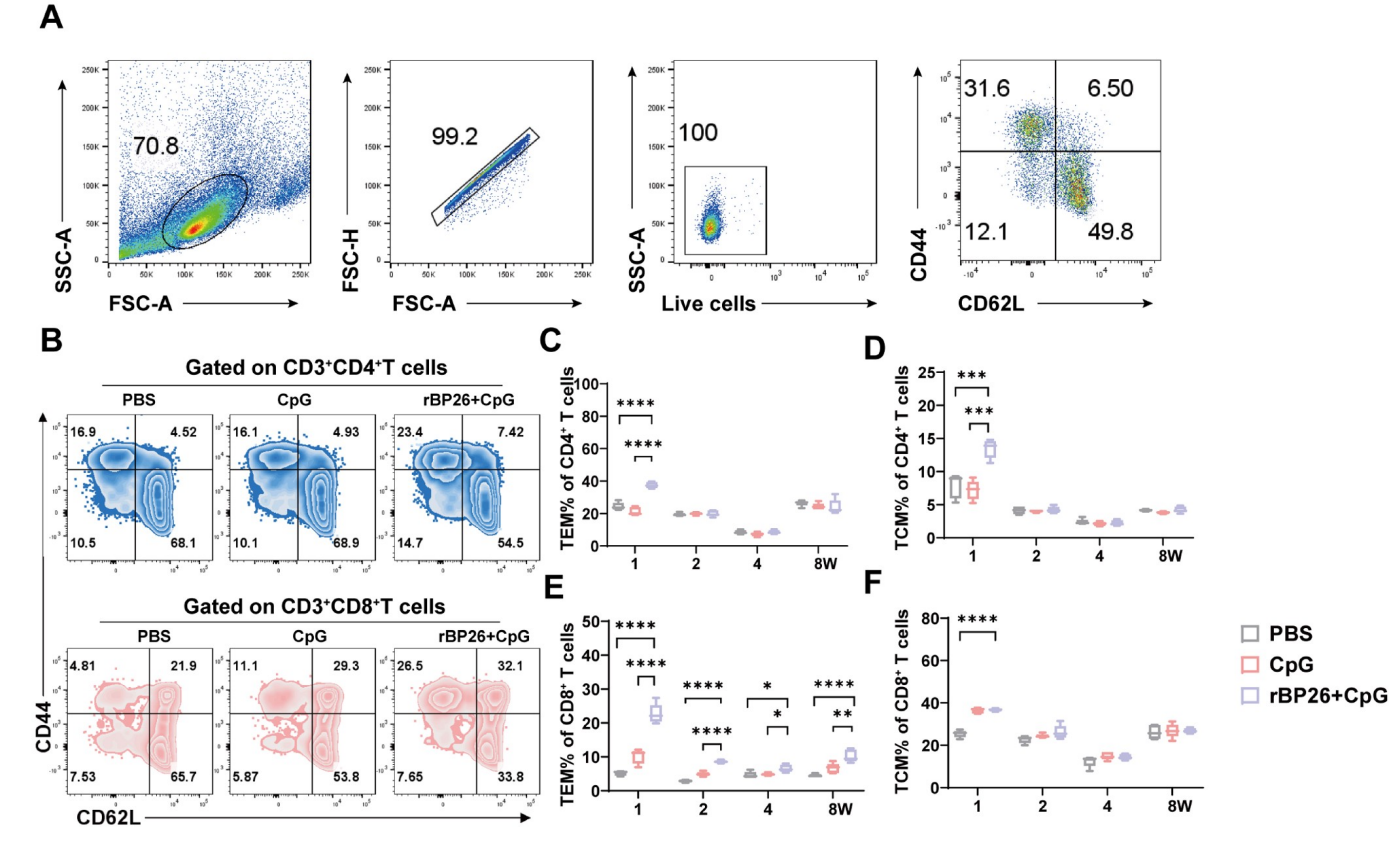
Figure 8 Memory phenotypes of CD4 ^+^ and CD8 ^+^ T cells Splenocytes were collected at 1, 2, 4 and 8 weeks after the last immunisation to determine the memory phenotype of CD4+ and CD8+ T cells. (A) The gating strategies for CD44+CD62- T cells and CD44+CD62+ T cells. (B) Representative contour plot analysis of gated CD4+ T and CD8+ T cells expressing CD44 and CD62L. Numbers of CD4+ T cells presenting the cell surface phenotype (C) CD44+CD62− T cells and (D) CD44+CD62+ T cells. Numbers of CD8+T cells presenting the cell surface phenotype (E) CD44+CD62− T cells and (F) CD44+CD62+ T cells. Data were collected from a sample size of 5 mice, and the error bars indicate the standard deviation. *P<0.05, **P<0.01, ***P<0.001, ****P<0.0001.

In addition, we conducted a comprehensive investigation into the differentiation patterns of CD4 ^+^ and CD8 ^+^ T cells following subcutaneous administration of rBP26+CpG. To achieve this goal, we employed a sorting strategy to identify central memory T cells (TCMs) and effector memory T cells (TEMs) ( Figure 8A,B).

The results revealed a significant increase in the frequency of CD44 ^+^ CD62L ^–^ TEM and CD44 ^+^ CD62L ^+^ TCM cells among the CD4 ^+^ T cells in the rBP26 + CpG group compared to that in the PBS and CpG groups one week after the final immunization ( Figure 8C,D). Furthermore, the induction of TEM within the CD8 ^+^ T-cell population in the rBP26+CpG group was significantly greater than that in the other two groups at weeks 1, 2, 4, and 8 following the final immunization ( Figure 8E). In contrast, the rate of TCM induction in the CD8 ^+^ T cell population was significantly higher than in the PBS groups only at week 1 after the last immunisation ( Figure 8F).

## Discussion

Brucellosis, a severe zoonotic disease, can be effectively controlled through vaccination. However, progress in the development of brucellosis vaccines has been slow, and human vaccines are not yet available. The challenge lies in developing a safe and effective vaccine against *Brucella*, a parthenogenous intracellular pathogen that mainly resides in macrophages. Protective immunity against brucellosis relies on cellular immune responses dominated by the Th1 subpopulation of T lymphocytes, which play a central role in combating infection [21]. Currently, vaccine development has entered the molecular vaccine stage, and recombinant protein vaccines show promise as safer, cheaper, and more suitable candidates for large-scale production than conventional vaccines. These recombinant protein vaccines have been shown to induce cell-mediated immune responses. Previous studies have demonstrated the immunogenicity and protective properties of *Brucella* OMPs, which are crucial for maintaining membrane integrity and selective permeability. As a result, additional studies have focused on using *Brucella* membrane proteins as subunit vaccines to provide protection against *Brucella* infection [ 22‒ 24]. Although many cell surface and intracellular components have been identified as potential factors for preventing *Brucella* infection, only low to moderate levels of protection have been achieved [ 9, 25].

In addition, previous research has focused primarily on OMPs [ 26, 27], whereas BP26 was initially identified as the primary protein responsible for the immune response in animals infected with *Brucella*. It was discovered to possess a distinctive functional structural domain known as “SIMPL,” which implies that BP26 may have a role in the innate immune response pathway [28]. This study aimed to further investigate the impact of BP26 on host immune responses and propose potential immune mechanisms. *Brucella* enters the host and persists within host macrophages, resulting in chronic infection [ 29, 30]. As the first line of defense against *Brucella*, macrophages play a crucial role in maintaining internal environmental stability and eliminating pathogenic microorganisms [31]. Macrophages (M0) employ various activation mechanisms to effectively combat pathogenic microorganisms and/or host mediators. These mechanisms equip them with either proinflammatory and bactericidal effects (M1 macrophages), which play a crucial role in humoral immunity and the proinflammatory response *in vivo* [32], or anti-inflammatory effects (M2 macrophages), which are primarily involved in curbing the inflammatory response and facilitating tissue repair [33]. Furthermore, studies conducted on animals have revealed notable disparities in macrophage subpopulations during different stages of *Brucella* infection. During the early/acute stage of infection, the presence of M1 macrophages fosters enhanced bactericidal capacity, while heightened expression of IFN-γ leads to a reduction in *Brucella* survival within the spleens of infected mice. In contrast, the later stages of infection are characterized by an increase in M2 macrophages, which prolongs the survival time of *Brucella* [34]. Numerous studies have substantiated the efficacy of modulating macrophage polarization. Our findings indicate that, compared to those in the PBS and CpG groups, the proportion of M1-type macrophages significantly increased, while the proportion of M2 macrophages notably decreased in the rBP26-immunized group. Additionally, the production of proinflammatory cytokines (TNF-α and IL-6), vital effectors mediating macrophage resistance against *Brucella* infection, was augmented in the rBP26-immunized group. These cytokines stimulate phagocytic lysosomal fusion events and the generation of antimicrobial effectors such as ROS, NO, and lysosomal enzymes [ 35, 36]. Thus, these *in vivo* experiments suggest that BP26 serves as a pivotal initiator of innate immunity, specifically activating macrophages to trigger antimicrobial effectors, thereby facilitating early control of infection.

Macrophages play a crucial role in both innate and adaptive immunity, acting as a vital link between the two. Upon receiving activation signals from antigen-presenting cells, initial CD4 ^+^ T cells differentiate into various helper T-cell subtypes, guided by the provided information and cytokines. Th1 cells secrete type 1 cytokines, such as IFN-γ, which are key cytokines in the immune response to *Brucella* infection. This cytokine not only protects the host during the early stages of infection but also stimulates the proliferation of CD8 ^+^ T cells. On the other hand, Th2 cells secrete type 2 cytokines, such as IL-4 [21], which contribute to the chronic infection caused by *Brucella* [37]. The results of this study, obtained through ELISA, ELISPOT, and flow cytometry analysis, demonstrated that immunization with rBP26 effectively stimulated the production of Th1-type cytokines, including IFN-γ, IL-2, and TNF-α, while Th2-type cytokines, such as IL-4, were not significantly affected compared to those in the PBS or CpG group. Furthermore, rBP26 also stimulated the production of IFN-γ by both CD4 ^+^ and CD8 ^+^ T cells. These findings suggest that rBP26 induces a Th1-biased T-cell response, which may contribute to the protective effects against *Brucella* infection [38]. Overall, these results provide further evidence that modulating macrophage polarization and promoting a Th1-type immune response, as achieved through rBP26 immunization, can effectively control *Brucella* infection. By activating macrophages and stimulating the production of antimicrobial effectors, rBP26 serves as a crucial initiator of innate immunity, facilitating the early control of infection. This study highlights the importance of macrophages and T-cell responses in the immune defense against *Brucella* and provides valuable insights for the development of novel therapeutic strategies.

Antibody-mediated humoral immune responses also play a crucial role in regulating the levels of circulating *Brucella* in the bloodstream of infected hosts. Compared to those in the PBS or CpG groups, the presence of rBP26-induced antibodies persisted for a longer duration, and the IgG titres were greater. Moreover, the production of both IgG1 and IgG2a antibodies increased. These observations suggest that rBP26 effectively induces Th1-type cellular immune responses and stimulates the production of associated antibodies [39]. Furthermore, our study revealed that the introduction of rBP26 resulted in an increase in CD69 and CD25 expression on CD4 ^+^ and CD8 ^+^ T cells after exposure to the homologous antigen for one day. The frequency of CD69 expression was notably greater in the rBP26 group than in the PBS and CpG groups. Additionally, rBP26 also facilitated the proliferation of specific CD4 ^+^ and CD8 ^+^ T cells. In conclusion, the changes in cytokine and antibody profiles, together with the enhanced activation and proliferation of CD4 ^+^ and CD8 ^+^ T cells in response to rBP26, collectively indicate the effectiveness of rBP26 in stimulating Th1-type cellular immune responses and associated antibody production [40].

Immune memory is an essential aspect of adaptive immune responses, and the formation and maintenance of memory cells are crucial for inducing long-lasting protective immunity [42]. While the production of cytokines and antibodies is associated with terminal differentiation and exhaustion, memory cells are responsible for controlling *Brucella* and other infections, manifested in the secondary response of the immune system to encountered antigens. Among memory cells, CD8 ^+^ T cells play a critical role in controlling pathogenic infections. Therefore, the generation of protective memory CD8 ^+^ T cells through vaccination is an attractive strategy for preventing and treating various human diseases. These memory CD8 ^+^ T cells enhance the host′s ability to defend against secondary infections [ 41‒ 45].

Given the importance of optimizing vaccine-induced immunity, it is crucial to determine the composition of protective memory CD8 ^+^ T-cell responses. In our study, we observed that, compared with the control treatment, vaccination with rBP26 induced both effector and central memory CD4 ^+^ T cells (CD4 ^+^ TEM and CD4 ^+^ TCM cells) as well as CD8 ^+^ T cells (CD8 ^+^ TEM and CD8 ^+^ TCM cells). Furthermore, effector memory CD8 ^+^ T cells were found to persist for up to 8 weeks after the last immunization. These findings suggest that the rBP26 vaccine promotes the development of durable immune memory, which is primarily dominated by effector memory CD8 ^+^ T cells. This further enhances our understanding of BP26-induced protective T-cell immunity and advances research on anti-brucellosis vaccines.

Our findings provide valuable insights into the immunological properties of rBP26. We observed that rBP26 induces M1 macrophage polarization, promoting Th1-type cellular immune responses. Additionally, rBP26 has been shown to stimulate lymphocyte activation and proliferation, resulting in long-lasting immune memory. These novel findings build upon previous basic immunological studies of rBP26 and offer significant contributions toward understanding the immune mechanisms involved in host clearance of *Brucella*. With these compelling results, further studies focusing on rBP26 as a recombinant protein vaccine hold great promise in the prevention of *Brucella* infections.
